# Determinants favoring weight regain after weight-loss therapy among postmenopausal women

**DOI:** 10.1038/s41598-020-74302-7

**Published:** 2020-10-19

**Authors:** Joanna Bajerska, Agata Chmurzynska, Agata Muzsik-Kazimierska, Edyta Mądry, Beata Pięta, Maciej Sobkowski, Jarosław Walkowiak

**Affiliations:** 1 Department of Human Nutrition and Dietetics, Faculty of Food Sciences and Nutrition, Poznań University of Life Sciences, Wojska Polskiego 31, 60-624 Poznan, Poland; 2 First Subdepartment of Pediatrics, Department of Pediatric Gastroenterology and Metabolism, Poznań University of Medical Sciences, Szpitalna 27/33, 60-572 Poznan, Poland; 3 Department of Maternal and Child Health, Poznań University of Medical Sciences, Polna 33, 60-535 Poznan, Poland

**Keywords:** Diseases, Medical research, Risk factors

## Abstract

Little is known about the factors affecting body weight-loss maintenance among postmenopausal women. We thus performed an analysis to identify some sociodemographic, physiological, and behavioral predictors of weight regain in a targeted subpopulation of women who had lost weight 1 year earlier. We also measured how eating behaviors and habits as well as physical activity pattern differ among successful and unsuccessful weight-loss maintainers over the trial. Sixty-four postmenopausal women were followed up for a year after dieting, and the successful and unsuccessful maintainers were identified. The regainers had regained an average of 4.9 kg of their lost body weight, while the maintainers had regained only 1.5 kg. Regainers had fewer years of education and lower initial body weight loss than maintainers. They also showed poor dietary adherence during dieting, and had unhealthy patterns of eating involving the avoidance of breakfast and a lower intake of nuts, seeds, and pulses, and a higher intake of sweets, biscuits, cakes, and pastries over time (excluding the dieting period). All the significant sociodemographic, physiological and behavioral variables differentiating regainers and maintainers before and after dieting were then examined as independent variables in a logistic regression model. The model showed that less weight reduction during dieting, higher disinhibition scores after dieting, and avoidance of breakfast before dieting were significant predictors of body weight regain in postmenopausal women. From a practical point of view, early identification of postmenopausal women who are at risk of regaining lost weight can allow health professionals to create behavioral and dietary supports to help prevent this. A regular schedule of follow-ups over at least the first year should be considered for them—including psychological and dietary intervention, if necessary. Since this sample study included only postmenopausal women, our findings are not generalizable to other populations.

## Introduction

It has been calculated that obesity is three times more common in postmenopausal women than in younger women^1^. Excessive body weight, especially in the form of visceral fat deposition among postmenopausal women, contributes to systemic inflammation and the development of metabolic syndrome, which in turn increases the risk of cardiovascular disease, diabetes, and mortality^2^. Although over half of women attempt to lose weight after menopause, the majority of these regain 30–50% of the lost weight over the following years, once again placing them at a higher risk of developing obesity-related diseases^3,4^. This statistics indicates that successful weight maintenance remains a challenge among the obese postmenopausal women, as well as in other obese individuals trying to keep lost weight off after dieting. There are various compensatory mechanisms that make it difficult to maintenance a new, lower weight^5–7^. One fundamental adaptations to weight loss is that the lower weight contributes to a decline in energy expended^6,8^. In particular, a decline in lean mass contributes to a lower resting metabolic rate (RMR). Moreover, after dieting physical activity (PA) will be less energetically expensive when moving smaller body mass^8^. During the weight-loss maintenance period, individuals thus need to be more active than during dieting^9,10^. Depletion in a fat mass reduces energy expenditure, primarily by altering metabolic efficiency via its role in homeostatic regulation (i.e., reduced leptin, insulin)^7,8^. Weight regain is also promoted by the suppression of postprandial fat oxidation^6^. Decreases in fat storage during dieting—by reductions in leptin levels and increases in ghrelin contribute to higher feelings of hunger and higher energy intake and storage^6^. It has also been suggested that weight regain may be associated with a disruption in sensitivity to these hormones^6^.

In order to keep weight off, individuals must at least continue to adhere to the behavior they adopted during dieting, in order to counteract the physiological adaptations associated with weight regain. However, this can be difficult to achieve when in an environment where palatable and energy-dense foods are readily available and sedentary behavior is common^8^. It could also be the case that losing less weight during dieting may lead to a total breakdown of eating controls, thus promoting weight regain^11,12^. On the other hand, eating restraint is known to increase with successful weight loss^13^. Losing less weight during dieting is associated with lower adherence to the prescribed diet and with a lesser ability to replace long-term pre-diet habits and behaviors by new ones that promote weight maintenance and overall health. In this matter, changes from an irregular meal rhythm to a more regular rhythm, including eating breakfast, has been identified as helpful in long-term weight loss and weight loss maintenance^13^. It has been postulated that some sociodemographic factors (e.g. lower level of education) may also contribute to the development of obesity and post-dieting weight regain^14,15^. Since marital status is associated with body weight, lowering its value^16^, it would be interesting to check whether marital status is associated with a predisposition to maintain or regain lost weight.

If weight maintenance strategies are to be improved, it is thus necessary to determine the factors that predispose to weight regain after dieting. Some studies have previously addressed this issue^17–20^. There is an association in formerly obese populations between weight regain after dieting and lower resting metabolic rate (RMR)^17^. In addition, decreases in dietary restraint and increases in dietary disinhibition have been found to be associated with weight regain over 10 years^18^. Finally, post-obese regainers experienced greater difficulty in continuing food and exercise behaviors during the follow-up period^19^, and the study conducted by Byrne et al. among obese women aged 20–60 years showed that the factors predisposed to regaining lost weight were associated with dissatisfaction with the weight achieved by dieting^20^. Yet there remains a great deficit in our knowledge of the factors that favor weight regain among postmenopausal women who lost weight 1 year earlier. Identifying these factors could reduce the gaps in knowledge and help create more effective supports assisting postmenopausal women to sustain their new weight and to manage obesity and its various comorbidities.

We thus performed an analysis to identify some of the predictors of weight regain in postmenopausal women who had lost weight a year earlier. To achieve this, we make use of pre-diet and post-diet factors associated with sociodemographic variables (age, age at final menstrual period, years of education, and marital status) physiological variables (body weight and RMR measured before dieting, changes in these variables after dieting, and proportion of body fat reduced after dieting), and behavioral variables (adherence to the diet during dieting, scores for restraint, disinhibition and hunger and physical activity pattern measured before and after dieting). We also measured how eating behaviors and habits as well as physical activity pattern differ among successful and unsuccessful weight-loss maintainers over the study trial.

## Methods

### Study design

**Figure 1:**
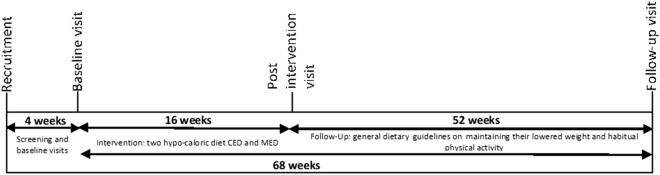
Timeline of the study.

This article presents 1-year follow-up data from a randomized clinical trial (DRKS00012958; https://www.drks.de/drks_web/), in which we evaluated and reported^21^ the effectiveness of two weight-loss dietary interventions differing in macronutrients—the hypocaloric Mediterranean diet (MED) and the hypocaloric Central European diet (CED)—on changes in body weight, fat mass, dietary adherence, and metabolic-syndrome-related indicators in postmenopausal women for 16 weeks. The trial lasted 68 weeks, and had a dietary intervention period of 16 weeks. It involved one baseline visit, control visits every 4 weeks from baseline up to week 16 of the study, and then a follow-up period lasting 52 weeks, during which there was only one, control visit (week 68). The timeline of the study is presented in Fig. 1.

One hundred and forty-four Caucasian nonsmoking centrally obese postmenopausal women (BMI 33.7 ± 0.4 kg/m^2^; age 60.5 ± 0.5 years) were recruited in 2014 through advertisements to participate in this study. We previously reported the details of the recruitment procedure, all the inclusion and exclusion criteria, and the composition of the study diets^21^. After receiving a clear explanation of the study, all subjects gave their written informed consent to participate. Justification of the sample size and of the randomization procedure, conducted in accordance with the CONSORT 2010 guidelines, were also previously reported^21^. The design of this study was approved by the local ethics committee at Poznań University of Medical Sciences (no. 603/14) in agreement with the Helsinki Declaration. On week sixteen of the study, the postmenopausal women who had completed the dietary intervention (n = 130) were discharged to the community with only general dietary guidelines on maintaining their lowered weight. Throughout the study period, they were also asked to maintain their habitual physical activity level. The women received no contact from study personnel until a year after the baseline period, on week 68 of the study, when they were contacted to assess changes in body weight and in eating behavior and habits, as well as to access physical activity measures.

**Figure 2:**
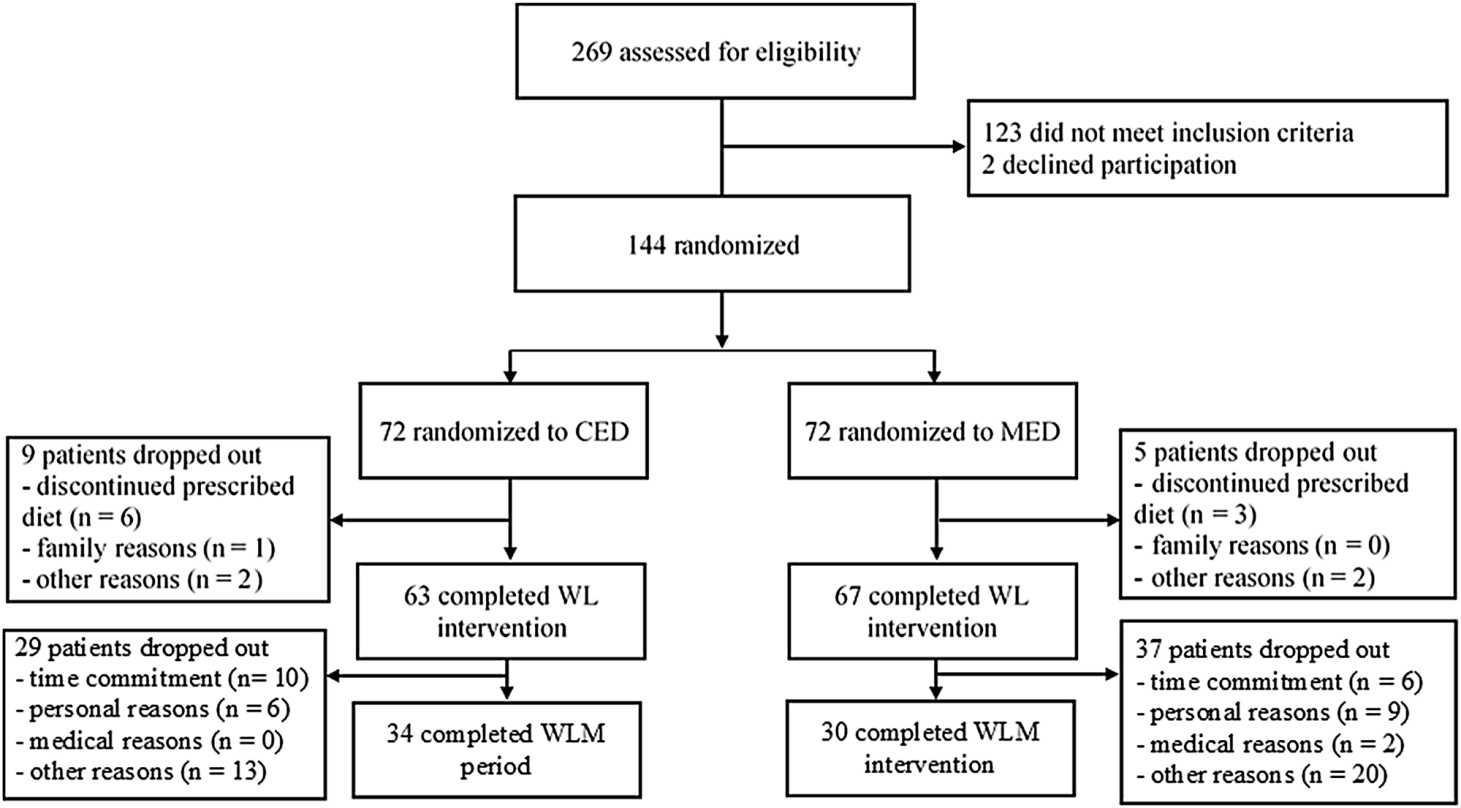
Flow chart. *CED* Central European diet, *MED* Mediterranean diet, *WL* Weight loss, *WLM* Weight-loss maintenance.

The follow-up data presented here uses only the results from the 64 participants who completed follow-up period. By the time of the 1-year follow-up, 66 participants (51% of the initial group) were lost due to time commitment reasons (n = 16), personal reasons (n = 15), medical reasons (n = 2), or unknown reasons (n = 33, Fig. 2). The number of women who were not able to maintain their lowered body weight was similar for both weight-loss intervention groups (CED = 34 and MED = 30). For the data presented here, body weight, and self-reported eating behavior measures (such as dietary restraint, disinhibition, and hunger) were collected at baseline after dieting and then at the 1-year follow-up. RMR and fat mass were collected before and after dieting. Information on dietary intake and the eating of breakfast was collected before dieting and at the 1-year follow-up. The self-reported PA pattern was assessed before and then the 1-year follow-up. Sociodemographic variables were collected from the participants upon their entry to the trial.

The small number of postmenopausal women attending the follow-up study (n = 64) translated into small sample sizes for each dietary intervention group, and therefore small statistical power. Because of this, both dietary groups were merged, though the type of weight-loss diet continued to be used as a potential confounder in the statistical analysis. To indicate the predictors associated with weight regain after 1-year of follow-up, participants were classed as successful weight-loss maintainers (those with at least 5% weight loss below their baseline weight at follow-up) or unsuccessful weight-loss maintainers (all others)^22^.

### Outcome measures

#### Sociodemographic factors

Sociodemographic variables (age, age at final menstrual period, years of education, and marital status) were collected from the participants at their entry to the trial.

### Physiological factors

We determined height using the standard protocol with an anthropometer (WPT 200.OC) and body weight with a Bod Pod scale (Cosmed, Rome, Italy). Body fat content was assessed using a dual-energy X-ray absorptiometry (DXA) scan. We measured RMR with indirect calorimetry using a canopy system and standard operating procedures (Quark RMR, Cosmed, Rome, Italy).

### Behavioral factors

The nutrition survey included the question “How many days per week do you eat breakfast?”; women who reported eating breakfast 5–7 days per week were categorized as breakfast eaters, while women who reported eating breakfast 4 or fewer days per week were categorized as breakfast skippers. Breakfast was defined as any meal eaten in the morning (6:00–9:00) consisting of any type of food, including milk. We evaluated eating behavior using the Three Factors Eating Questionnaire (TFEQ)^23^. This is a 51-item scale measuring three domains of eating behavior (dietary restraint scale, dietary disinhibition scale, and hunger scale), consisting of 36 closed questions with a forced true/false response and 15 Likert-rated items. This measure has high internal consistency (Cronbach’s alpha 0.82 to 0.90) for the three scales in samples of dieters and free eaters^23^.

Dietary intake was assessed using three-day dietary recall covering 2 weekdays and 1 weekend day. Subjects were asked to keep a food diary recording all food and drink consumed, using household measures to quantify serving sizes. Dietetyk (Jumar, Poznań, Poland), a dietary analysis software program, was used to calculate energy and selected macronutrients and micronutrients. We have previously described adherence to the prescribed diet^21^ using a Mahalanobis distance equation^24^ Physical activity (PA) level was assessed using the short version of the International Physical Activity Questionnaire (IPAQ-SF)^25^, which elicits information on physical activity over the ‘last seven-day’ period. We have previously described the procedure for assessing PA patterns^21^. This questionnaire was assessed in a pilot study for validity and reliability, according to the international scheme^26^. According to the work of Craig et al. on the international reliability and validity study of the IPAQ instruments, the criterion validity had a median rho of about 0.30^27^.

### Statistical analysis

We grouped the baseline variables of age, age at final menstrual period, years of education, and marital status together as *sociodemographic factors*. We took body weight, RMR before dieting, and the changes in these variables after dieting—as well as the proportion of body fat reduction during the diet—together *as physiological factors*. In turn, dietary adherence during the diet period, scores for restraint, disinhibition, and hunger before and after dieting, as well as one-year follow-up and physical activity pattern measured before and after dieting were grouped together as *behavioral factors*. A two-sample *t*-test was used to compare the two groups for normally distributed continuous variables. We used a χ^2^ test to compare nominal data. The effects of group and time were then tested using the general linear model (GLM) with an adjustment for PA and the type of weight-loss diet. Where necessary, we carried out post-hoc comparisons between treatment groups using the Bonferroni correction. The significant variables (*p* < 0.05) in the univariate analysis were then examined as independent variables in direct multiple logistic regression, with unsuccessful weight-loss maintenance as the dependent variable. We successively removed the least informative covariates were from the model in a backward stepwise elimination procedure.

The study was powered so as to detect a between-group difference in body weight change of ± 3.0 kg at a 1-year follow-up. We considered *p* < 0.05 to be statistically significant. The data were analyzed using Statistica software (StatSoft, Tulsa, OK, USA).

## Results

Postmenopausal women responding to the follow-up had lost about 1.9 kg more weight after dieting than the nonresponders. Moreover, there were no differences in any of the sociodemographic variables in Table 1 between participants responding to the follow-up study and the nonresponders (data not shown). The further analysis deals only with postmenopausal women responding to the follow-up study, divided into successful and unsuccessful weight-loss maintainers. The sociodemographic, physiological, and behavioral characteristics of the postmenopausal women are shown in Table 1.

**Table 1:** Sociodemographic, physiological, and behavioral characteristics of the postmenopausal women.

Variables	Maintainers N = 31	Regainers N = 33	*p* value*^*
**Sociodemographic factors**			
Age (years)	61.0 ± 1.0	60.0 ± 1.0	0.112
Age at final menstrual period (years)	49.5 ± 1.0	50.5 ± 0.5	0.372
Education (years of education)	15.0 ± 0.5	13.0 ± 1.0	0.032
Marital status, married (n/%)	24/77	23/70	0.485
**Physiological factors**			
Body weight before dieting (kg)	83.9 ± 2.0	83.8 ± 2.1	0.962
Changes of body weight after dieting (kg)	− 10.7 ± 0.6	− 6.5 ± 0.5	< 0.001
Changes of body weight at follow-up (kg)	1.5 ± 0.5	4.9 ± 0.5	< 0.001
Proportion of fat mass reduction to weight loss (%)	82.8 ± 3.9	91.5 ± 4.1	0.128
RMR at baseline (kcal/day)	1489.5 ± 24.0	1550.6 ± 27.7	0.102
Changes of RMR after dieting (kcal/d)	− 131.6 ± 9.7	− 123.5 ± 12.0	0.599
**Behavioral factors**			
Dietary adherence to weight-loss intervention (scores)	1.89 ± 0.08	2.19 ± 0.09	0.017
*Breakfast eating, yes (n/%)*			
Before dieting	23/74	14/42	0.010
One-year follow-up	27/87	18/54.5	0.004
*Energy intake (kcal/d)*			0.053^$^
Before dieting	1968.1 ± 58.6	1892.7 ± 55.4	
One-year follow-up	1719.3 ± 40.0	1884.8 ± 90.0	
*Fruit intake (g/d)*			0.253^$^
Before dieting	226.3 ± 26.3	283.0 ± 48.7	
One-year follow-up	227.5 ± 24.2	218.5 ± 22.1	
*Vegetable intake (g/d)*			0.565^$^
Before dieting	370.0 ± 29.4	285.1 ± 29.4	
One-year follow-up	422.3 ± 31.4	300.5 ± 26.5	
*Whole cereal intake (g/d)*			0.102^$^
Before dieting	106.2 ± 11.8	73.2 ± 9.3	
One-year follow-up	147.5 ± 10.8	142.2 ± 11.9	
*Nut, seed, and pulse intake (g/d)*			0.023^$^
Before dieting	5.9 ± 2.3	0.3 ± 0.3	
One-year follow-up	32.8 ± 8.0^a^	6.9 ± 3.5^b^	
*Sweet, biscuit, cake, and pastry intake (g/d)*			0.021^$^
Before dieting	68.2 ± 6.5	60.4 ± 16.2	
One-year follow-up	35.9 ± 6.4^a^	77.4 ± 16.7^b^	
*PA before dieting*			
< 600 MET/min/week (n/%)	10/32	12/36.5	
600–1499 MET/min/week (n/%)	17/55	16/48.5	0.877
≥ 1500 MET/min/week (n/%)	4/13	5/15	
*PA one-year follow-up*			
< 600 MET/min/week (n/%)	9/29	11/33	
600–1499 MET/min/week (n/%)	16/52	18/55	0.720
≥ 1500 MET/min/week (n/%)	6/19	4/12	

Quantitative data are shown as means ± SEMs; Qualitative data are shown as n/%; ^*p* value of *t*-test or χ^2^ test. ^$^Results from the general linear models on the effects of group and time, adjusted for physical activity and assigned weight-loss diet; Different superscript letters indicate statistically significant differences, *p* < 0.05. *MET* metabolic equivalent, *RMR* resting metabolic rate, *PA* physical activity.

### Sociodemographic predictors of weight regain

Regainers had significantly (*p* = 0.032) fewer years of education than maintainers (13.0 ± 1.0 years vs. 15.0 ± 0.5 years). There were no other differences in sociodemographic variables between the groups (Table 1).

### Physiological predictors of weight regain

At the 1-year follow-up, maintainers had regained 1.5 ± 0.5 kg of their lost body weight, while the regainers had regained 4.9 ± 0.5 kg (*p* < 0.001); regainers also had significantly (*p* < 0.001) lower initial body weight loss (6.5 ± 0.5 kg) than maintainers (10.7 ± 0.6 kg).

### Behavioral predictors of weight regain

**Figure 3:**
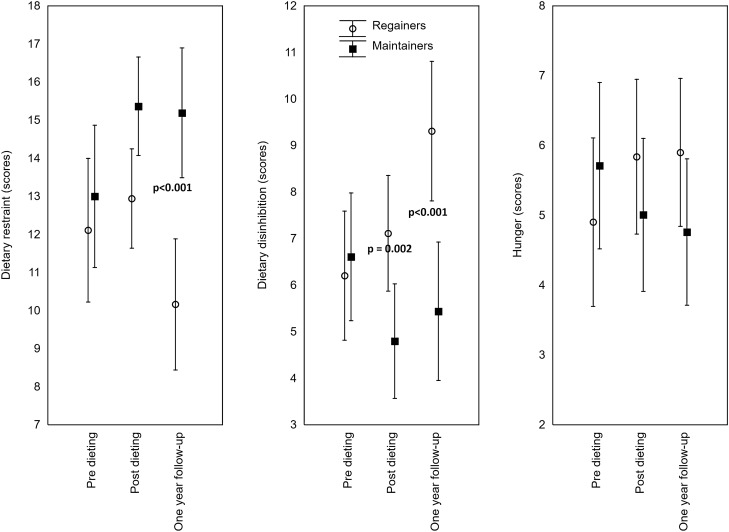
Mean dietary restraint, disinhibition, and hunger scores over the study period. Results from the GLM adjusted for physical activity and the type of weight-loss diet. Post-hoc comparisons between treatments groups were performed using the Bonferroni criterion.

With regard to behavioral factors, dietary adherence across dietary interventions was significantly lower (*p* = 0.017) among regainers (2.19 ± 0.09) than among maintainers (1.89 ± 0.08). Over time, regainers displayed significantly lower dietary restraint (*p* = 0.018), defined as a tendency to consciously restrict or control food intake. A post-hoc analysis revealed that there was a significant difference (*p* < 0.001) in restraint scores between regainers and maintainers at the 1-year follow-up. Over time, regainers also presented a significantly higher disinhibition pattern (*p* < 0.001), defined as a tendency to overeat in the presence of palatable foods or other stimuli. A post-hoc analysis revealed that there were significant differences in disinhibition scores between regainers and maintainers after dieting (*p* = 0.002) and at 1-year follow-up (*p* < 0.001, Fig. 3). Before dieting and 1-year follow-up maintainers and regainers presented similar physical activity patterns (Table 1).

Before dieting, fewer weight regainers than maintainers reported eating breakfast (*p* = 0.010). This difference between the groups was also similar at the 1-year follow-up (*p* = 0.004).

Over time (excluding the dietary intervention phase), and controlling for potential confounders, regainers reported a lower daily intake of nuts, seeds, and pulses (*p* = 0.023). Post-hoc analysis revealed that there were significant differences (*p* < 0.05) in the daily intake of these food items between regainers and maintainers at the 1-year follow-up. Regainers reported a higher consumption of sweets, biscuits, cakes, and pastries (*p* = 0.021) over time than did weight maintainers. Post-hoc analysis revealed that there were significant differences (*p* < 0.05) in the daily intake of these food items between regainers and maintainers at the 1-year follow-up.

In the next step, all significant sociodemographic, physiological, and behavioral variables differentiating regainers from maintainers before and after dieting were examined as independent variables in a logistic regression model. This demonstrated (Table 2) that weight relapse was associated with a smaller initial body weight loss after dieting (odds ratio [OR] 1.839, 95%CI 1.335–2.157, *p* < 0.001) and with the ability to eat disinhibitedly after dieting (OR 1.551, 95%CI 1.335–2.535, *p* = 0.009). Weight regain was also associated with a pattern of skipping breakfast (OR 6.345, 95%CI 1.191–33.809, *p* = 0.030), as recorded before the weight-loss intervention.

**Table 2:** Results of multivariate logistic regression analysis to predict weight regain.

Independent variable	β	SE β	Wald χ^2^	OR (95% CI)	*p* value
Initial weight loss (kg)	0.609	0.164	13.881	1.839 (1.335–2.157)	< 0.001
Disinhibited eating pattern after dieting	0.439	0.168	6.811	1.551 (1.335–2.535)	0.009
Skipping breakfast before dieting	1.848	0.854	4.685	6.345 (1.919–33.809)	0.030

Odds ratios (OR) and 95% confidence intervals (CI) are presented. Results from multiple logistic regression, with unsuccessful weight-loss maintenance as the dependent variable. The least informative covariates were successively removed from the model in a backward stepwise elimination procedure. Adjusted to physical activity and diet assigned during weight-loss phase.

## Discussion

In our analysis, weight-loss maintenance after initial weight loss was successful for 31 participants (48%) and unsuccessful for 33 (52%). The regainers put on an average of 4.9 kg of their lost weight, while maintainers put on only 1.5 kg. Our results are opposed to those of the meta-analysis of Franz et al. (2007), who noted that as much as two-thirds of lost body weight was maintained in the first year after weight-loss treatment^28^. However, the studies included in this meta-analysis were not aimed at postmenopausal women, which suggests to us that some of the specific physiological, behavioral, or sociodemographic variables may be associated with a failure to maintain the lower weight in that study populations. In the next stage of our analysis, we thus identified those variables that were significantly different between the regainers and maintainers. In terms of sociodemographic variables, the regainers had significantly fewer years of education than did the maintainers. With regard to physiological variables, regainers had less initial body weight loss after dieting than did the maintainers. Finally, taking into account behavioral variables, regainers had tendency to omit breakfast and showed poor adherence to their assigned diets. Those who regained more weight after 1 year of follow-up were less restrained eaters than the weight maintainers, and were more likely to be disinhibited eaters, both after dieting and at the 1-year follow-up. One-year follow-up maintainers and regainers presented similar physical activity patterns. Regainers showed a tendency towards lower intakes of nuts, seeds, and pulses, and higher intakes of sweets, biscuits, cakes, and pastries over time (excluding weight-loss phase) than the maintainers.

However, the main aim of the present study was to identify which variables most effectively predict the magnitude of weight regain in postmenopausal women. The significant pre-diet and post-diet sociodemographic, physiological, and behavioral variables recognized between regainers and maintainers were therefore examined as independent variables in a multiple logistic regression. We found that the factor that most predicted weight regain within 1 year after dieting was small initial weight loss. The second major factor predicting weight regain was disinhibition pattern recognized after dieting; the final predictor was a pattern of skipping breakfast before dieting. These factors in our model have been well documented for their association with risk of weight regain; however, to our knowledge, such associations have never been reported for the subpopulation of postmenopausal women. It should thus be highlighted that our study addresses existing gaps within the literature regarding the effect of specific factors on weight gain among postmenopausal women.

The meta-analysis of Anderson et al. (2001) also confirmed that people who lost more weight during weight-loss therapy maintained significantly more weight loss in the long-term than those who lost less weight^29^. Less weight loss during dieting is associated with a drop in the participant’s satisfaction with the results^30^ and, when an “all or nothing” attitude exists, this lack of satisfaction may lead to a subsequent total breakdown of eating controls and weight regain. People who lost less weight during dieting were also considered to be less motivated and less engaged in long-term changes in their dietary behavior^14,31^. Indeed, regainers showed a tendency towards the greater intake of sweets, biscuits, cakes, and pastries over time (excluding the weight-loss phase) than did maintainers. A reduced intake of particular food types, including sweets, has been associated with better maintenance of weight loss^13^. Moreover, regainers from our study more frequently omitted breakfast. Smaller initial weight loss may also reflect worse compliance with the prescribed dietary treatment^13^. Indeed, in our study, adherence to diets was significantly poorer in regainers than in maintainers. The initial weight lost is not the only important factor affecting weight relapse; another one is the percentage of weight loss as fat, especially in middle-aged populations. Vogels and Westerterp-Plantenga (2007) reported that the percentage weight regained after 2 years of a very low-calorie diet was associated with the percent of body fat lost during the treatment^32^. Studies of changes in body composition following weight-loss therapy in middle-aged obese dieters showed a strong tendency to return to the starting weight^33^, which can be explained as the absence of the fat-free mass-sparing effect^31,34^. Nonetheless, in our study, both maintainers and regainers had favorable body composition changes, as their weight losses were 82.8% and 91.5% as fat, respectively. Changes in RMR after the weight-loss therapy also did not differ between the groups. The physical activity patterns did not differ between the groups either.

Certain behavioral factors, including disinhibited eating, have been identified in the literature as contributing to weight regain^35^. Karlsson et al. (1994) suggested that a higher level of disinhibition may not be a very potent factor during the weight-loss phase—when the effect of disinhibition is attenuated by a simultaneously high level of restraint—but may gain significance during weight maintenance^36^. Moreover, disinhibition is also associated with less healthful food choices, which contribute to subsequent weight regain and poorer health^14,37^. Greaves et al. (2005) stressed in their systematic review that people who regained the weight they had lost were able to return to old food-choice patterns and feel “stuck or used to some type of food or diet”^38^. As demonstration of this, our study showed that dietary patterns after dieting did not remain consistent with the recommendations given before it; rather, there was a gradual onset of undesirable dietary habits developing into what had been observed prior to dieting, such as lower intake of nuts, seeds, and pulses—all food items whose consumption has been recognized as beneficial in promoting weight loss and maintenance^39,40^, on account of their unique fiber, protein, and fat composition—and higher intake of sweets, biscuits, cakes, and pastries, which are recognized as harmful to healthy body weight. Moreover, regainers tended to avoid breakfast. Jakubowicz et al. (2017) observed that skipping breakfast adversely affects clock and clock-controlled gene expression, and is correlated with increased postprandial glycemic response and future weight gain^41^. From a behavioral point of view, the pattern of breakfast avoidance by regainers in our study might be explained by a false perception that reducing the number of meals helps to lose more weight or to maintain reduced weight^42^. In fact, daily breakfast consumption is a common eating behavior among people who have maintained their weight after weight-loss management^14,42,43^, Kruseman et al. (2017) found that the breakfast-eating pattern was similar among those maintaining weight loss and those maintaining a stable normal weight, concluding that individuals have to find their own eating rhythm that allows them to maintain their weight^44^. On the other hand, a recently published systematic review and meta-analysis of randomized controlled trials concluded that skipping breakfast, rather than eating it, might help people lose weight; this all suggests that eating patterns and their effect on sustaining healthy weight constitute a multithreaded issue^45^. Since habitual patterns of eating may remain relatively stable over time among middle-aged women^13^, there is a need to create a more intense or prolonged dietary intervention in this subpopulation, in order to remodel these less healthy eating patterns.

We identified several limitations to this study. The first is that the number of participants who were available at the follow-up was relatively small, but statistical differences were found even after adjusting for potential confounders. We can thus conclude that these findings are representative of the study group. Moreover, those who failed to return to the follow-up study lost less weight after dieting than did the responders. They might therefore have perceived the follow-up as a part of the failed treatment. This is a particular issue for dietary programs, where a lack of success may be perceived as a personal failure, rather than in a pharmacological intervention, where failure might be viewed as due to the lack of efficacy of the drug^47^. This sample also included only postmenopausal women, which prevents us from generalizing to other populations. However, we can also report some strengths of this research. In particular, our study only included postmenopausal women who were not repeat dieters; this behavior is associated with changes in metabolism that increase metabolic efficiency and decrease energy demands.

## Conclusion

We found that some eating behaviors of regainers did not change over time when the study was conducted. Regainers had tendencies to omit breakfast and to intake smaller quantities of nuts, seeds, and pulses, and higher intakes of sweets than maintainers. We identified three main predictors of future weight regain in postmenopausal women: The data suggest that less weight loss during dieting, a propensity for disinhibited eating after dieting, and skipping breakfast before dieting all were significant predictors of future weight regain in the target population.

From a practical point of view, early identification of postmenopausal women who are at risk of regaining lost weight can allow health professionals to create behavioral and dietary supports to help prevent them. For these patients, a regular follow-up schedule over at least the first year should be considered with psychological interventions (such as practicing stimulus control techniques) and dietary interventions (such as mindful eating) made available when necessary.
